# Pseudohyponatremia: A Potentially Dangerous Laboratory Artifact

**DOI:** 10.7759/cureus.90901

**Published:** 2025-08-24

**Authors:** Zahra Amiri, Justin Peterson

**Affiliations:** 1 Internal Medicine, University of California Los Angeles David Geffen School of Medicine, Los Angeles, USA

**Keywords:** cholestasis, hypercholesterolemia, hyponatremia, lipoprotein x, pseudohyponatremia

## Abstract

Pseudohyponatremia is a rare laboratory artifact that inaccurately reports a falsely low serum sodium in the presence of excess lipids and/or proteins. Pseudohyponatremia secondary to excess lipids such as lipoprotein X is less common than hypertriglyceridemia or paraproteinemia and occurs in the setting of cholestasis. Here, we present a case of pseudohyponatremia due to suspected elevated serum lipoprotein X levels. Direct ion measurement of serum sodium levels confirmed normal serum sodium levels and the diagnosis of pseudohyponatremia in the presence of vastly elevated cholesterol levels. Prompt recognition of this clinical entity avoided unnecessary and potentially harmful interventions to “correct” the serum sodium levels.

## Introduction

Pseudohyponatremia is an uncommon but important clinical entity characterized by a falsely low serum sodium concentration in the setting of normal serum osmolality [1]. This artifact results from the displacement of plasma water by excessive lipids or proteins, particularly when sodium is measured using indirect ion-selective electrodes. In indirect ion measurement, a serum sample is diluted with a fixed volume of diluent prior to measurement of the ions, and this calculation is based on an assumption about the percent that solid components, mainly proteins and lipids, occupy in the sample. Thus, a larger-than-expected amount of proteins and/or lipids in the sample will be unaccounted for by this assumption and result in a miscalculation of the concentration of sodium ions in the sample. Conversely, direct ion measurement does not involve diluting a sample or making assumptions on the amount of protein and lipids in the sample, and thus sodium ion measurement is unaffected by large quantities of these solid components in a serum sample analyzed by this method [1]. While most cases are associated with hypertriglyceridemia or paraproteinemia, pseudohyponatremia secondary to hypercholesterolemia, especially due to the presence of lipoprotein X (Lp-X), is rare [2]. Lp-X is an abnormal lipoprotein that accumulates from significant biliary stasis and can severely distort the accuracy of both lipid panels and electrolyte measurements [3,4].

Failure to recognize pseudohyponatremia can lead to unnecessary and potentially harmful interventions to inappropriately “correct” a “low” serum sodium with treatments such as hypertonic saline administration and ICU admissions. We present the case of a 70-year-old woman with severe cholestasis, profound hypercholesterolemia, and pseudohyponatremia secondary to suspected Lp-X accumulation. Her case highlights the diagnostic challenges and importance of considering pseudohyponatremia in patients with significant liver dysfunction and abnormal sodium values.

## Case presentation

A 70-year-old female was admitted to the hospital for evaluation of jaundice and elevated liver enzymes after being transferred from a recuperative care facility, where she had been placed following a previous hospitalization for suicidal ideation. Two months prior to the current presentation, she had presented to an outside hospital with a constellation of symptoms, including jaundice, elevated liver enzymes, blurry vision in the left upper visual field, and suicidal ideation attributed to significant psychosocial stressors. After spending one day at the recuperative care facility, she again endorsed suicidal ideation along with generalized weakness, which prompted her transfer to our institution.

Upon presentation, the patient was afebrile with a heart rate of 75 beats per minute, respiratory rate of 18 breaths per minute, blood pressure of 158/109 mmHg, and oxygen saturation of 99% on room air. She appeared jaundiced but was in no acute distress. Her psychiatric evaluation revealed a calm, cooperative demeanor with linear thought processes and denial of current suicidal ideation. Neurologic examination was non-focal and cranial nerves were intact. Pulmonary, cardiovascular, and abdominal exams were unremarkable. Initial laboratory work-up is below (Table 1).

**Table 1:** Initial Laboratory Values C-ANCA: cytoplasmic anti-neutrophil cytoplasmic antibodies, P-ANCA: perinuclear anti-neutrophil cytoplasmic antibodies

Laboratory Test	Result	Reference Range
White Blood Cell Count	7.59 ×10E3/uL	4.16-9.95 x10E3/uL
Hemoglobin	9.8 g/dL	11.6-15.2 g/dl
Platelets	390 ×10E3/uL	143-398 x10E3/uL
Sodium	114 mmol/L	135 - 146 mmol/L
Potassium	3.6 mmol/L	3.6 - 5.3 mmol/L
Chloride	83 mmol/L	96 - 106 mmol/L
Bicarbonate	23 mmol/L	20 - 30 mmol/L
Blood Urea Nitrogen (BUN)	15 mg/dL	7 - 22 mg/dL
Creatinine	0.42 mg/dL	0.6 - 1.3 mg/dL
Aspartate Aminotransferase (AST)	112 U/L	13 - 62 U/L
Alanine Aminotransferase (ALT)	98 U/L	8 - 70 U/L
Alkaline Phosphatase	3,404 U/L	37 - 133 U/L
Total Bilirubin	8.2 mg/dL	0.1 - 1.2 mg/dL
Albumin	2.0 g/dL	3.9 - 5.0 g/dL
Total Protein	5.4 g/dL	6.1 - 8.2 g/dL
Hepatitis B Surface Antigen	Nonreactive	No reference range
Hepatitis B Surface Antibody	Negative	< 8.5 mIU/mL
Hepatitis B Core Antibody	Nonreactive	No reference range
Hepatitis C Antibody	Nonreactive	No reference range
Human Immunodeficiency Virus (HIV) Antigen / Antibody - 4th Generation	Nonreactive	No reference range
Antinuclear Antibody	< 1:40 titer	< 1:40 titer
Mitochondrial Antibody	< 1:20 titer	< 1:20 titer
Smooth Muscle Antibody	< 1:20 titer	< 1:20 titer
C-ANCA	< 1:20 titer	< 1:20 titer
P-ANCA	< 1:20 titer	< 1:20 titer
Proteinase-3 Antibody	< 20.0 CU	< 20.0 CU
Myeloperoxidase Antibody	< 20.0 CU	< 20.0 CU
Alpha-1-Antitrypsin Antibody	183 mg/dL	90 - 200 mg/dL
Ceruloplasmin	35 mg /dL	14-45 mg/dL

The patient previously had a measured serum sodium of 134 mmol/L the day prior. Due to perceived profound acute hyponatremia, she was transferred overnight to the intensive care unit for further evaluation and management. In the intensive care unit, she reported mild fatigue but denied confusion, nausea, or headache. She noted recent polyuria with large unmeasured urine volumes but denied excessive thirst. She also endorsed decreased appetite. On examination, the patient was noted to have normal mentation and orientation. The patient's gait was not assessed. A 250 mL bolus of lactated Ringer's solution was given for her perceived hyponatremia and her measured serum sodium improved by 1 mmol/L on recheck an hour and a half later. A second 250 mL bolus of lactated Ringer's solution was administered with a subsequent decrease of 2 mmol/L of her measured serum sodium checked two hours after the previous check. Salt tablets were ordered but were never administered. Nephrology was consulted to evaluate and recommend treatment for the patient's low measured serum sodium. The patient's work-up for the etiology of her hyponatremia is summarized below (Table 2).

**Table 2:** Subsequent Laboratory Values *(by direct ion measurement, six hours after initial serum sodium measurement by indirect ion methods)

Laboratory Test	Result	Reference Range
Serum Osmolality	295 mOsm/kg	275 - 295 mOsm/kg
Urine Osmolality	568 mOsm/kg	No reference range
Urine Sodium	61 mmol/L	No reference range
Serum Uric Acid	2.4 mg/dL	2.9 - 7 mg/dL
Serum Cortisol (AM)	13.2 mcg/dL	8 - 25 mcg/dL
Thyroid Stimulating Hormone	3.8 mcIU/mL	0.3 - 4.7 mcIU/mL
Total Cholesterol	2,464 mg/dL	No reference range
High-Density Lipoprotein (HDL)	4 mg/dL	> 50 mg/dL
Low-Density Lipoprotein	110 mg/dL	< 100 mg/dL
Triglycerides	337 mg/dL	< 150 mg/dL
Serum Sodium*	136 mmol/L	135 - 146 mmol/L

Given the patient was relatively asymptomatic for having a "drop" in her serum sodium from 134 mmol/L to 114 mmol/L in a 24-hour period, as well as her significant cholestasis and normal serum osmolality, pseudohyponatremia was suspected. Direct ion-specific sodium measurement confirmed a corrected serum sodium of 136 mmol/L six hours after her initial serum sodium was measured by indirect methods. A nuclear magnetic resonance lipid profile was sent to further investigate for the confirmation of the presence of elevated serum lipoprotein X levels in the setting of severe cholestasis, but the sample was deemed insufficient for analysis. Unfortunately, the patient was discharged from the hospital prior to this result and a second sample was not obtained to confirm the identity of the lipid(s) mainly responsible for the patient's severe hypercholesterolemia. The patient was started on icosapent ethyl and on recheck three months later, her serum total cholesterol level was 394 mg/dL and serum sodium by indirect ion measurement was 137 mmol/L.

## Discussion

This case illustrates a rare but important diagnostic challenge: pseudohyponatremia due to lipoprotein X accumulation in the setting of severe cholestasis. Although the patient’s initial sodium level was measured as being critically low (114 mmol/L), her lack of associated symptoms for a perceived 20 mmol/L drop in her serum sodium over 24 hours and normal measured serum osmolality fortunately led to the correct and prompt identification of pseudohyponatremia. Confirmation of a normal serum sodium by direct ion-selective measurement, as well as normalization of serum sodium values by indirect measurement following an improvement in the patient's cholesterol level, supported the diagnosis of pseudohyponatremia.

Pseudohyponatremia occurs when nonaqueous plasma components - typically lipids or proteins - displace plasma water, causing indirect assays to underestimate serum sodium. When a sample is measured using indirect measurement of ions, the serum sample is diluted with a set volume of diluent prior to measurement, based on the assumption that serum samples all have a similar amount of nonaqueous particles. When the level of nonaqueous particles is unbeknownst to be elevated, due to excess proteins or lipids, the aqueous phase will be a proportionally smaller amount of the sample. Thus, dilution of the sample with a fixed amount of diluent will result in a less concentrated aqueous phase of the sample, which is where the sodium ions are measured [1]. While hypertriglyceridemia and paraproteinemia are more commonly implicated, Lp-X-induced pseudohyponatremia is far less frequently encountered but increasingly recognized in patients with cholestatic liver disease and biliary stasis [3-6]. Unlike hypertriglyceridemia, elevated Lp-X levels will not cause a serum sample to appear lipemic, which adds to the diagnostic challenge of this entity [6].

Lp-X is an abnormal lipoprotein rich in unesterified cholesterol and phospholipids and lacking apolipoprotein B, which prevents clearance via low-density lipoprotein (LDL) receptors. Instead, Lp-X accumulates in the plasma during cholestasis when bile lipids reflux into circulation and subsequently bind to albumin [4,7-9]. Not only does it interfere with sodium measurements by indirect methods but it can also alter lipid panels, often yielding extremely elevated total cholesterol with disproportionately low high-density lipoprotein (HDL) and LDL values [10].

Our patient exhibited these hallmark features: cholestasis, severe hypercholesterolemia (>2,400 mg/dL), low HDL (<4 mg/dL), and absence of typical hyponatremia symptoms, prompting suspicion for raised serum Lp-X. Though confirmatory testing for Lp-X via lipoprotein electrophoresis was unfortunately inconclusive due to an inadequate sample, the clinical and biochemical context aligned with Lp-X-associated pseudohyponatremia reported in similar cases [4-6].

Early recognition of this entity is essential. Unwarranted treatment of apparent hyponatremia with fluid restriction, salt tablets, or hypertonic saline may lead to serious neurological complications or even death [1,11]. Moreover, unnecessary ICU admissions and costly evaluations can be avoided with timely use of direct sodium measurements and awareness of Lp-X in the differential diagnosis of hyponatremia in patients with cholestasis [1].

## Conclusions

This case underscores the importance of integrating clinical presentation with laboratory interpretation, particularly in the context of liver dysfunction. Knowing the method of serum sodium measurement is also paramount to knowing whether a patient's serum sodium result could be subject to this laboratory artifact. When extreme hypercholesterolemia coexists with unexpectedly low serum sodium, often with a normal serum osmolality, Lp-X-mediated pseudohyponatremia should be high on the differential and confirmatory testing should be performed immediately to avoid adverse outcomes.
